# Supplementary material for: Causal interplay between lactose intolerance and gut microbiota: a combined bidirectional Mendelian randomization and in vivo validation study
Source: Front Nutr. 2026 Jun 1;13:1803337. doi: 10.3389/fnut.2026.1803337 (PMC13265576; doi:10.3389/fnut.2026.1803337)
Supplement: Supplementary file 1 [file Data_Sheet_1.zip › supplementary materials/Forward/forest plot/ebi-a-GCST90027816.finngen_R12_E4_LACTONAS.pdf]

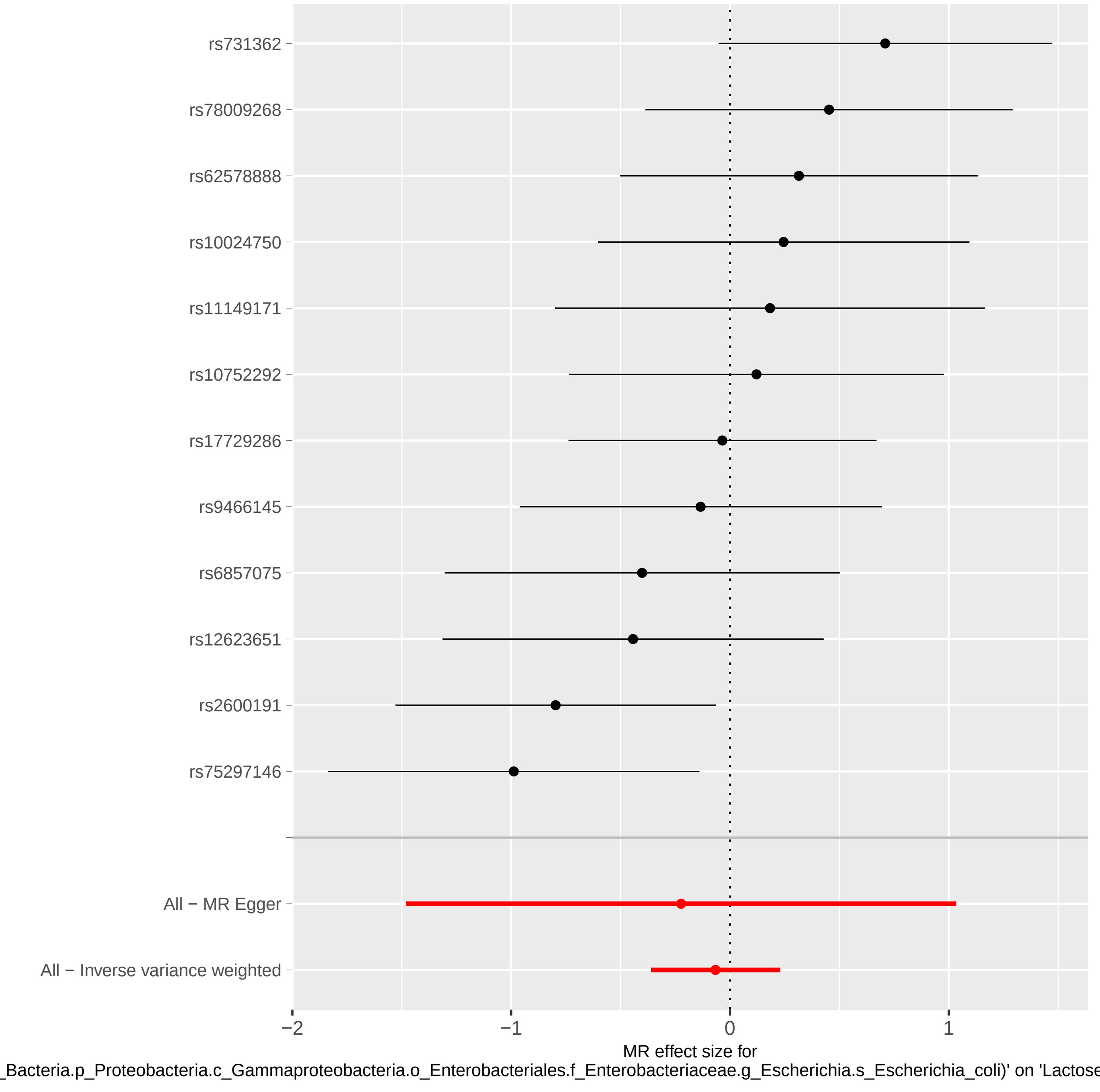
rs731362
rs78009268
rs62578888
rs10024750
rs11149171
rs10752292
rs17729286
rs9466145
rs6857075
rs12623651
rs2600191
rs75297146
All − MR Egger
All − Inverse variance weighted
−2
−1
0
1
MR effect size for
_Bacteria.p_Proteobacteria.c_Gammaproteobacteria.o_Enterobacteriales.f_Enterobacteriaceae.g_Escherichia.s_Escherichia_coli)' on 'Lactose
